# Exploring the future of GM technology in sustainable local food systems in Colombia

**DOI:** 10.3389/fgeed.2023.1181811

**Published:** 2023-06-30

**Authors:** Néstor Julián Cárdenas Pardo, Dolly Esperanza Rodriguez Robayo, John Cristhian Fernandez Lizarazo, Diego Camilo Peña-Quemba, Erica McGale

**Affiliations:** ^1^ Utopía, Universidad de La Salle, Yopal, Colombia; ^2^ Faculty of Natural Sciences and Engineering, Fundación Universitaria de San Gil, UNISANGIL, Yopal, Colombia; ^3^ Department of Ecology and Evolution, University of Lausanne, Lausanne, Switzerland

**Keywords:** genetically modified (GM) plants, GM technology, local agriculture, Colombia, rural education, tropical agriculture

## Abstract

The security of Earth’s food systems is challenged by shifting regional climates. While agricultural processes are disrupted by climate change, they also play a large role in contributing to destabilizing greenhouse gases. Finding new strategies to increase yields while decreasing agricultural environmental impacts is essential. Tropical agriculture is particularly susceptible to climate change: local, smallholder farming, which provides a majority of the food supply, is high risk and has limited adaptation capacity. Rapid, inexpensive, intuitive solutions are needed, like the implementation of genetically modified (GM) crops. In the Latin American tropics, high awareness and acceptance of GM technologies, opportunities to test GM crops as part of local agricultural educations, and their known economic benefits, support their use. However, this is not all that is needed for the future of GM technologies in these areas: GM implementation must also consider environmental and social sustainability, which can be unique to a locality. Primarily from the perspective of its educators, the potential of a rural Colombian university in driving GM implementation is explored, including the role of this type of university in producing agricultural engineers who can innovate with GM to meet regionally-dependent environmental and cultural needs that could increase their sustainability.

## Introduction

The unprecedented acceleration of climate change in our current era, the Anthropocene, must be imminently addressed (Lamb et al., 2021). Agriculture, defined as plant and animal systems that produce services for humans, is a particularly large contributor to climate change (Lamb et al., 2021; Tubiello et al., 2021; Zurek et al., 2022). Agriculture produces greenhouse gases (GHGs) through the energy needs of, for example, fertilizer production (Bennetzen et al., 2016; Richards et al., 2019). Simultaneously, increased GHGs are among the predominant elements limiting agricultural productivity (Tyczewska et al., 2018). Abiotic and biotic factors are exacerbated by a changing climate. These include occurrences of extreme temperatures, unpredictable precipitation amounts and frequencies, weather disasters, and unexpected life history shifts for herbivore and pollinator populations. Agricultural plants must simultaneously face below-ground, altered soil fertility as well as shift their plant physiology to face new above-ground challenges, all which require energy that can be redirected away from their agricultural output (Montesinos-Navarro et al., 2020).

Increasing amounts of academic publications report, discuss, and attempt to find strategies to address agriculture’s contribution to climate change (Malhi et al., 2021). Proposals range from reducing GHGs based on creating “climate-smart” soils that recapture carbon released during plant agriculture (Lipper et al., 2014), and extend to helping farmers adopt climate-resilient methodologies, including regenerative agriculture, agroforestry, soil management, and water harvesting (Altieri and Nicholls, 2017). The use of new techniques within current, established systems can mitigate climate change to an extent (Adam et al., 2020). However, some recent studies point out limitations: for example, applying soil conservation methods can take a significant number of years before yield profits are generated, if at all, which does not respond to the immediate needs of farmers (Thierfelder et al., 2017; El Chami et al., 2022). Long implementations are also incompatible with current rapid shifts in regional climates caused by overall global warming (Singh et al., 2017).

Where the adoption of any new agricultural method by, for example, Latin American farmers, is already particularly risky, lack of guaranteed benefits and the length of transition scenarios may additionally explain low adoption of environmentally sustainable agricultural methods (Vignola et al., 2022). The easiest and most rapid changes, like increasing the use of input products (e.g., fertilizers, herbicides), or implementing double cropping (two growth cycles of plants in order to ensure yields despite environmental changes), are preferable, but definitively unsustainable (Chen et al., 2022): they more rapidly affect soil health, which continues to add to the frailty of future agricultural systems in terms of resilience and production. To prevent these problems, agricultural systems, and especially those that may be most vulnerable like those in the tropics and/or global south, need inexpensive, rapid, and ideally, environmentally sustainable, solutions to the consequences of climate change.

Genetically modified (GM) crop use has great potential in Latin American food systems. While the use of GM plants in pharmaceutics, disease prevention, and bioremediation has not been contentious, the adoption of such plants in food systems has been gradual, despite the remarkable benefits (Sikora and Rzymski, 2021). As of 2022, implementations of GM crops worldwide were reported to have increased crop yields by 22%, which is 10% more than by conventional crop improvements over the same time period (Klümper and Qaim, 2014; Caradus, 2022). Farmers using GM crops were shown to have 68% more profits on average, with considerably higher gains (i.e., 60% more) occurring in developing versus developed countries (Klümper and Qaim, 2014). Contrary to some preconceptions (Marris, 2001; Cui and Shoemaker, 2018; Lynas et al., 2022), there is no concrete evidence that any GM crop material, in itself, has ever caused declines in mental or physical attributes of animals or humans (Shen et al., 2022). Academics and other professionals exhibit a consensus for the continued development of new GM innovations to improve plant agriculture (Tang et al., 2009), and the public may be converging towards this as well (Evanega et al., 2022; Mohd Saad et al., 2022). Like with all new agricultural implementations, they still require proper testing and systems in place to guarantee this, but these should not prevent them from being able to aid farmers in need (Datta et al., 2007; Prost et al., 2023).

It is perhaps most urgent to explore the integration of GM crops in tropical agriculture because the tropics have and will be disproportionately affected by climate change: due to a historical stability of tropical climates, plant plasticity may be severely decreased in these ecosystems, which make them less resilient to change (Trisos et al., 2020). Lack of resiliency in tropical and subtropical food systems have even larger implications for the farmers and the communities they feed; in these regions, the majority of food is produced by local, smallholder farms (Jones and Thornton, 2003; Timmons Roberts, 2009). In Latin America, these producers account for up to 67% of the food supply, depending on the country (Barrientos-Fuentes and Torrico-Albino, 2014). Taking action now to find and promote technologies like GM that can stabilize and sustain agricultural production, as well as promote resiliency and sustainability, will be particularly impactful. This is especially true for smallholder agricultural systems, which have the fewest adaptation possibilities (Zhou L. et al., 2022).

## Implementation of GM technology in Latin America

Europeans and North Americans are characterized as more familiar with GM technologies than others around the world (Woźniak-Gientka et al., 2022). Interestingly, however, only 21% of Europeans have heard about GM technology (Ewa et al., 2022). Generally, European populations are high in anti-GM sentiment (62.5% of the GM-knowledgeable public; Marris, 2001) and low in arable land use for GM crops (0.1% of arable land; Ichim, 2019; 2021). In the United States, less than 50% of people who know about GM plants are against them (Sikora and Rzymski, 2021), and 47.8% of arable land is planted with GM crops (ISAAA Brief No. 54, 2018; FAO, 2022). Clearly, public opinion is perhaps not the lone predictor of GM crop implementation. Information access and education, together with consumer beliefs and openness, play major roles in the acceptance of new technologies applied to food (Aleksejeva, 2012; Sikora and Rzymski, 2021; Woźniak-Gientka et al., 2022).

In 1992, 31 years after the Americas had begun using GM organisms in agriculture (Rangel, 2015), there were still objections to this technology in Latin America, driven by the concept of bioethical precaution (Gatica-Arias, 2020). The incipient acceptance of GM organisms in Latin America occurred through the education system (Zhou Y. et al., 2022), starting from high school students (Occelli and Valeiras, 2021) and continuing to the academic sector (Román Collazo et al., 2022). At this time, technical, agricultural schools also began making positive impacts in GM knowledge and awareness (Florek-Łuszczki et al., 2016). In the past 50 years, the support and growth of the agricultural education sector, both municipal and rural, has brought the newest technologies, including GM, into discussions had by students of all backgrounds across Latin America. This enables young people in many Latin American countries to have larger roles in the future of their countries’ agricultural systems (Fernández Lizarazo et al., 2020a; Cabrera Cabrera et al., 2020). Today, 44% of arable land in Latin America contains GM crops, making this continent the fastest growing implementor of new GM crop land area (Woźniak-Gientka et al., 2022).

## Learning by doing, teaching by demonstrating, and the rural reach of Utopía’s students

As much as agricultural education has improved across Latin America, it is still majorly limited in rural settings. In Colombia, this is due to inaccessibility of most rural regions to the construction of quality educational equipment (laboratories, internet networks, electrical and water installations, etc.), to networks of consumable providers to supply educational materials, and to sufficiently-trained professors that can provide a quality education (Carrero Arango and González Rodríguez, 2017; Gaviria, 2017; Galvis et al., 2021). Most members of rural populations look to pursue agricultural education in the nearest large city, but this requires means for travel and subsistence in the city, which is often inaccessible, not subsidized by the educational institution, and not supported by the government (Guzmán Rincón et al., 2021). Apart from lack of financial means, family obligations can discourage pursuits of higher education when coming from rural areas (Gaviria, 2017).

The department of Casanare in Colombia, containing rural plains to the east of the Andes, presents these challenges to its residents, in addition to general inequality, poverty, lack of safety and low productivity of land (IDEAM et al., 2017; Céspedes, 2021). These are shared with other regions in Colombia, as well as with rural areas across Latin America (Stampini et al., 2016). However, Casanare contains a rural university that may be tackling these challenges in a unique manner. For 13 years, La Salle University has been committed to rural development by providing a high level of education to young people anywhere in the country, from local areas to the farthest and poorest areas in Colombia; students come to its university campus, called “Utopía” (Flechas Hernandez and Molano Camargo, 2019; Flechas Hernández et al., 2020). These diverse students come to train as plant and livestock agricultural engineers (i.e., agroengineers; Table 1).

**TABLE 1:** Gender and regional (Colombia) demographics of La Salle student cohorts from 2013 to 2022.The Orinoquía region is where Casanare and Utopía campus (outside of Yopal), are situated. Amazonas = Amazon region; Andes = Andean region; Caribe = Caribbean Coast region; Orinoquía = Orinoco River basin; Pacífico = Pacific Coast region.

Year	2013 (%)	2014 (%)	2015 (%)	2016 (%)	2017 (%)	2018 (%)	2019 (%)	2021 (%)	2022 (%)
**% Woman**	26.7	30.4	23.7	32.7	48.7	42.9	47.3	19.2	50.0
**% from Amazonas**	28.3	19.6	18.4	14.3	0.0	6.1	0.0	0.0	17.9
**% from Andes**	33.3	15.2	23.7	46.9	35.9	34.7	36.4	40.4	16.1
**% from Caribe**	11.7	10.9	26.3	6.2	28.2	22.4	18.2	28.8	21.4
**% from Orinoquía**	20.0	36.9	28.9	22.4	12.8	28.6	39.9	3.9	37.5
**% from Pacífico**	6.7	17.4	2.7	10.2	23.1	8.2	5.5	26.9	7.1

La Salle University employs pedagogical tools that promote methodology around “learning by doing and teaching by demonstrating” (Fernández Lizarazo and Peña Venegas, 2012; Fernández Lizarazo et al., 2020b). This methodology generates a synchrony between the classroom where the theory is presented, and the laboratory and field, where the theory is applied. This synchrony begins on campus with the “productive practice”: teachers and students first carry out applied research in the agricultural production system of the Orinoco River basin (“Orinoquía” from Table 1, within which Casanare is located). They tackle local limits to production including low soil fertility (acidic, nutrient poor soils) and a bimodal rainfall regime that creates the local flooded savannah ecosystem (IDEAM et al., 2017). These limitations allow the generation of research ideas to improve agricultural production under limited conditions. The students are additionally given contact with national and international universities (e.g., University of Lausanne, New Mexico State University, Universidad Nacional de Colombia), research entities (e.g., Agrosavia, CIAT), and unions (e.g., Fedearroz, Fedecacao), among others, in order to explore and study solutions to agricultural problems.

After this experience on campus, students are challenged to conduct a “productive project,” where in their last academic year, they return to their home regions to implement an academic (research) and agricultural (productive) extension of their education; they now have to apply their knowledge to the edaphoclimatic conditions, the system of commercialization, and the existing technologies of their home region in Colombia (Cárdenas Pardo et al., 2019; 2021; Flechas Hernández et al., 2020). Different from the Orinoco River basin, in the Colombian Pacific region, students will face the highest annual rainfall (6-7 m annually) in addition to highly acidic soils (IDEAM et al., 2017; Quinto-Mosquera and Moreno, 2017). In the Caribbean region they will face climate extremes - humid and dry forests, wetlands and deserts - all which share eroded soils with low soil organic matter (Quintero-Angel and Ospina-Salazar, 2022). The Andean region has a bimodal rainfall regime and a topography of steep slopes that result in sheet erosion (Quintero-Angel and Ospina-Salazar, 2022) and the Amazon region is an ecosystem rich in biodiversity that is highly susceptible to degradation when inadequate agricultural and livestock practices interrupt conserved land cover (Murillo-Sandoval et al., 2023). This diversity of regions and ecosystems in Colombia also determines the country’s agrobiodiversity: even commercialized crops are adapted over time to each region and the particularities of local food systems, an additional consideration for La Salle students.

The reality of local agricultural implementation introduces a new consideration as well for students: they need to harmonize the integration of new techniques and scientific principles acquired on campus with local, indigenous, and traditional knowledge (Cárdenas Pardo et al., 2019). Agroengineers across rural areas must manage preconceptions including preference to wild or “natural” foods grown without yield- and resistance-aids (i.e., fertilizers, herbicides, pesticides; Raza et al., 2021), and incorporate this into the pressing, but often opposing, need for more productive and resilient agriculture. Some of these preconceptions might encourage an avoidance of GM crops, within the guise that these are incompatible with more “natural” techniques such as the use of wild ancestors of crops or locally-adapted and traditional varieties. However, GM implementation can potentially support these values. Several research groups already study the application of GM in wild ancestors of crops as a way to minimally change them to increase their production or resilience; this maintains the evolutionary strength and plasticity of these plants, their histories, and other traditional uses, while also helping them to be competent in the face of unprecedented climate shifts (Indu et al., 2022; Petereit et al., 2022). Single GM modifications are very small, and could be considered equivalent to mutations that would occur naturally in these wild ancestors, or local varieties, over time. Knowing this, the Utopían students can be a bridge between the knowledge and application of GM crop technology and the local preferences and needs of rural, agricultural communities.

### The extended potential of teaching GM technologies on Utopía campus

Students of Utopía are trained to carry out lab and field trials framed in research activities, along with public and private companies, universities, or small producers looking to improve their productions in a sustainable way. Adoption success of new technology requires, among the most important aspects, appropriate testing (hypothesis-driven), which then includes follow-through tests and analysis, resulting in strategic and informed guidance (Westaway et al., 2023); the education of the implementors is often more important even than their available assets (Goodwin et al., 2022). When an agricultural leader can be both a farm manager as well as an advisor for such testing and adapting, this is an excellent advantage, especially in rural areas where it may be otherwise impossible to recruit partners and experts in the implementation and adaptation of new technologies.

Utopía students are able to develop innovative solutions from common and local materials. For example, a recent group of students and teachers developed a digital solar dryer for drying different types of seeds on Utopía campus. Surprisingly, this simple equipment had not yet been invented an adapted technology to rural needs, and is therefore now being patented (Patent submitted in Colombia). To get future leaders capable to advice, test, and adapt new technologies, La Salle teaches students to be resourceful and think critically: on other occasions when they conducted bioassays with inoculums of arbuscular mycorrhizal fungi (AMF) and saw results in increased cassava production (Ceballos et al., 2013), students immediately began to analyze the availability of this biotechnological application in Colombia. They found that there was no local solution for reliable production of this inoculum (i.e., viable spores, scalable, distributed), and this remains an ongoing problem (Salomon et al., 2022). Immediately, this became of interest as a potential future industry for these agroengineers.

**FIGURE 1:**
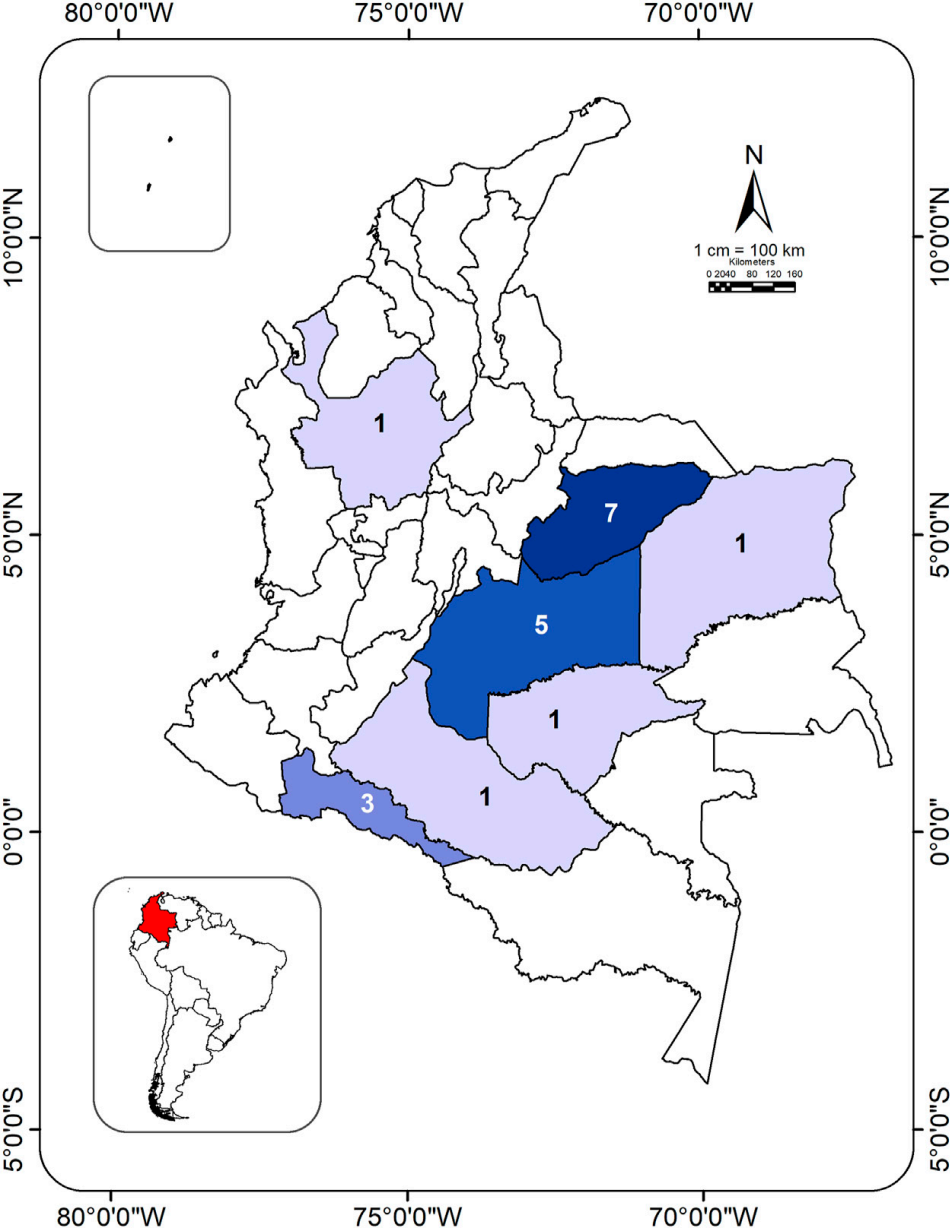
Distribution of productive projects by Utopía students, over the time period of 2011 to 2021, that specifically implemented the use of genetically modified (GM) corn. These represent only a portion of the productive projects, where others did not use GM crops, or did not use corn (Supplementary Table S1). Numbers of productive projects per department are shown. Departments having larger numbers of projects are portrayed in a darker shade of purple. For reference, the country of Colombia is localized in an inset map of South America (red). The shaded department most to the north in Colombia spans two biomes: those of the Caribe and the Andes. The rest of the departments span from the Amazonas (south-west) to the Orinoquían regions (west). Amazonas = Amazon region, Andes = Andean region, Caribe = Caribbean Coast region, Orinoquía = Orinoco River basin, Pacífico = Pacific Coast region.

La Salle students are aware of GM plant technologies and their economic, environmental, and social potentials in vulnerable tropical agricultural systems. Past students have already sown GM corn seeds (among other types of GM crops) with resistance to glyphosate herbicides, insects, or plant diseases, in both experiments on campus as well as in their productive project regions (e.g., Díaz Narváez, 2019; Casanova Linares, 2020; Gonzalez Mora, 2020; Navarro Holguín, 2020; Figure 1). Additionally, the students have access to GM technologies. Sources for production of new GM lines in corn and other crops are available in Colombia and in Latin America in general: 60% of the largest worldwide producers of GM plants are in Brazil, Argentina and Paraguay (Keiper and Atanassova, 2022). Still, most GM lines produced in Colombia use technology that were first developed in other areas (Brookes, 2020). In order to match regional needs, including the personalization of technologies to the effects of climate change on those regions, students of agricultural school systems like that of La Salle could become leaders in finding, creating, studying, and implementing new GM technologies more suitable to the environmental and cultural concerns of their home regions.

Students of Utopía become aware through their education that, for example, there does not yet exist a GM crop that can help plants tolerate, resist, or potentially alter highly acidic soils (Zheng, 2010; Magalhaes et al., 2018). Corrective applications are the current solution for this issue in Colombian regions (e.g., lime applications), as well as the application of fertilizers to address nutrient-poor soils, both which are carbon heavy to produce (Bennetzen et al., 2016). New GM technologies could include plants that tolerate high acidity, and reduce the production and application of carbon-costly products. This would also allow regional environments to maintain more of their natural characteristics, even when used for agricultural production. Along those lines, new GM plants might be made to more readily recruit AMF already present in Colombian soils; these mutualistic fungi known to help deliver plants with higher nutritional and water supplies despite deficiencies in soil (Huey et al., 2020) may also have potential in helping plants tolerate high acidic conditions (Alotaibi et al., 2021). The recruitment of local AMF(s) would also eliminate the need to create a local industry for AMF inoculum production.

New types of GM plants could also focus on increasing soil organic carbon through their increased transport of photosynthates to the soil (Gougoulias et al., 2014). Much like the Golden Rice GM lines produced to tackle widespread Vitamin A deficiency in India, this would involve optimizing the production of a molecule that most plants already have the potential to make, by performing genetic engineering in that molecular pathway (Paine et al., 2005). Classic regimes of mutation and subsequent evolution cause genes and enzyme structures to change, which alters their efficiency. Conventional breeding can mix and match these parts of molecular pathways to make more or less efficient combinations. GM simply recruits the most efficient forms of genes and implements them all at once, allowing the plant to optimize production in that pathway. Increasing soil organic carbon could add to the longevity and sustainability of arable land systems.

The resources to find genes to target with GM technology may not be available to agroengineers like those attending La Salle University. However, students can pull from single-gene and transcriptional manipulation studies that are already published in order to make suggestions for local developments of new GM lines. In addition to the research above that could inspire GM lines for acidity-tolerance or beneficial photosynthate exudation, there may be even more options for GM utility like in facilitating function at a community level. As it is known that plants have often been conventionally selected to tolerate increasing plant density, useful genes to modify, perhaps even in varieties that are less bred, may be those that enable higher cooperation among plants. Cooperation does not necessarily need to rely on interspecific mixes of plants (as from the well-known concepts of biodiversity to increase productivity and resiliency; resumed in Worm and Duffy, 2003). Intraspecific mixtures of plants that each contain differences at only single alleles, or even in single genes, can increase population yields in the field, and/or alter resistance to certain biotic factors (McGale et al., 2020; Montazeaud et al., 2022; Wuest et al., 2022). GM technology based on these results could replace intercropping strategies, which can be inefficient through complicated management and harvesting of multiple crops (Huss et al., 2022), while also bringing more options for creating resilience and stability in agricultural populations and surrounding plant communities. Regardless of the potential of these ideas, even well-known GM technologies can have very different regional dependencies and yield outcomes; *Bt* GM lines were shown to have yield benefits depending on the herbivore loads they were facing (McGale et al., 2018). Testing these new technologies is essential, and the agroengineers of La Salle are aware of this, and have begun to gain practice through their productive projects (Figure 1).

## Discussion

A system of training agroengineers like that in the Utopía campus of La Salle, paired with an openness for GM technologies seen growing in Colombia, sets up a location which could be ideal for rapid and impactful implementation of GM crops where they may be needed the most. These crops will likely not only help farmers produce more yields despite unique regional climate changes, bringing more economic stability, but also generally contribute to sustainable agriculture through reductions of economically and environmentally‐costly products as well as of expansions or intensifications of current agricultural systems (Arora and Montenegro, 2011; Tubiello et al., 2021; Murillo-Sandoval et al., 2023). GM implementations are not necessarily incompatible with the traditions of local farmers in using local or wild cultivars; GM may aid in preserving more “natural” cultivars by enabling them to resist dramatic climate shifts through an acceleration of natural adaptation. Finally, GM technology coming from and tested in the country where they will be implemented is not only more useful, but also an extension of economic sustainability.

Many existing and suggested GM technologies are reductionist, targeting “simple” issues within agroecosystems that limit crop productivity, like the ability of the plant to resist one or two factors (e.g., pesticides, pests, or diseases; Vanloqueren and Baret, 2009). As part of the implementation of GM technologies, it is important to integrate GM that may alter plant interactions among themselves and their surrounding ecosystem, which can unlock a potential for engineered plants to be a better fit for their regional ecosystems and climates, at the community level. This can reduce individual plant energy invested in responding to unexpected environmental factors instead of in yield. Example GM technologies of this sort could increase interactions with soil and soil communities, where altering a single allele (or functional single gene) frequency in a population might create a more synergetic population- or community-level effect. With more compatible plant-microbiome feedbacks, perhaps a better soil legacy may be produced, which could maintain the quality and production capacity of arable land. It is known that the alteration of a single plant metabolomic pathway, potentially through the modification of a single gene, can beneficially cascade through multiple trophic levels, from soil organisms to herbivore populations (Papantoniou et al., 2021; Sivaprakasam Padmanaban et al., 2022). The biodiverse, and varied, ecosystems of Colombia could offer a nice setting for some of the first, to our knowledge, integrations of such GM technologies, in the context of fast and sustainable solutions for local, smallholder systems.

Education provides opportunities and perspectives for a responsible participation in society, including the evaluation of information and decision-making (Aikenhead, 2003). Science education prepares people to manage complex relationships between science, technology, society, and environmental transformation (Occelli and Valeiras, 2021). Suggesting rural Colombian educational programs for the progress of GM technological implementation, and especially a program like the one at Utopía that integrates science education into agroengineering, is the first step, but action towards this is more difficult. Luckily, there are indications that Colombia, and Latin America, may already be set up to welcome the future of GM technologies. Already, conversations about GM technological solutions are more prevalent in Latin America than, for instance, on the African continent (Lynas et al., 2022), despite both having high dependence on local, smallholder food systems in susceptible tropical regions. Generally, academic reviews originating from research institutions in the global south equally, if not slightly more frequently, suggest GM technology as a pro-climate agricultural solution when compared to those originating from research institutions in the global north (Table 2). This reflects also the support from the academic sector in GM technology implementation in the global south.

**TABLE 2:** Absolute numbers and percentage breakdowns of academically-published, peer-reviewed review articles, published between January 2022 to February 2023, which fall under the search category “pro-climate agricultural adaptations” (*n* = 110). Raw data available in Supplementary Table S2. GM = genetically modified.

Majority authors representing	Amount	Mention GM	Do not mention GM	% of all mentioning GM
**Global north institutions** (% of global north institutions)	49	18 (36.7%)	31 (63.3%)	50
**Global south institutions** (% of global south institutions)	44	18 (40.9%)	26 (59.1%)	50
**Articles irrelevant to the search**	17			
**Total**	110			

It is true that common agricultural practices can and have already reduced the contribution of agriculture to, as well as the consequences on agriculture from, climate change (Sultan and Gaetani, 2016; Adam et al., 2020). However, yield changes that GM technologies have brought dwarf yield-optimizing efforts from conventional methods over the same time periods (Klümper and Qaim, 2014; Caradus, 2022). In Colombia, GM technologies have already reduced the application of insecticides and pesticides by 19 percent and have been especially effective in vulnerable, rural regions (Klümper and Qaim, 2014; Brookes, 2022). In general, GM technology can be enacted over a smaller time period, at a smaller cost, than conventional methods (El Chami et al., 2022; Woźniak-Gientka et al., 2022). In Colombian rural campuses like Utopía, GM methods can be integrated into the education of future agricultural leaders that can discuss, apply and analyze these solutions, in their local regions, where attention to new technologies is and will be especially important in the future and will help to preserve economic, ecological, social (i.e., cultural) resources. Attention to and integration of GM technologies in the training of future agricultural leaders, especially in tropical regions of the global south like those found in Colombia, are immensely important to consider in the preservation of world resources, and human life, in the face of climate change.

## Data Availability

The original contributions presented in the study are included in the article/Supplementary Material, further inquiries can be directed to the corresponding authors.

## Supplementary material

Supplementary Table S1 Summary table of Utopía student cohort data, between 2011 to 2021, on the productive projects they conducted. This includes the regions where they were conducted, the type of crop(s) they used, and whether the crops were genetically modified (GM) or not.

Supplementary Table S2 Summary table of academically-published, peer-reviewed review articles published from January 2022 to February 2023 that resulted from a Web of Science search for “pro-climate agricultural adaptation” (n = 110). This includes the author names, article title, journal title, publication month, publication year, other publication information, whether the article mentions genetically modified (GM) organisms and whether the article was published by a majority of authors at a global north or global south research institution. Some articles resulting from the search were considered “not applicable”, including articles only addressing pro-climate agricultural adaptations to animals or to urban landscapes. These were not considered for their GM mentions or author representations. Studies considered as mentioning GM had to be explicit in doing so. The use of the terms “biotechnology”, “stacking” and “tailored” could refer to conventional and GM methods, and were marked as the former as a default; therefore, the results tend to be on the conservative side in terms of claiming GM mentions. Finally, GM mentions do not imply that the mentions were in a positive, nor a negative light; it sufficed for the technology to be mentioned in the article for it to be marked.
